# Abnormal auditory neural activity in individuals with spinal muscular atrophy

**DOI:** 10.1093/braincomms/fcag281

**Published:** 2026-07-17

**Authors:** Gary Rance, Eppie M Yiu

**Affiliations:** Department of Audiology and Speech Pathology, The University of Melbourne, Parkville, VIC 3052, Australia; Department of Neurology, Royal Children’s Hospital, Parkville, VIC 3052, Australia; Neuroscience Research, Murdoch Children’s Research Institute, Parkville, VIC 3052, Australia; Department of Paediatrics, The University of Melbourne, Parkville, VIC 3052, Australia

**Keywords:** spinal muscular atrophy, hearing, auditory neural, binaural

## Abstract

Spinal muscular atrophy (SMA) is an autosomal recessive disorder characterized by hypotonia, progressive muscle weakness and atrophy caused by progressive loss of motor neurons in the spinal cord and lower cranial nerve nuclei. The disease has also been associated with sensory neuropathies affecting the visual, somatosensory and auditory pathways. This case-control study investigated auditory neural function and perceptual ability in children and young adults with SMA. Twenty individuals with genetically confirmed SMA of varying severity (Types 1–3) participated. Sixteen children/young adults aged 6–20 years and 16 age-, gender- and hearing-level matched controls underwent a battery of peripheral and central auditory assessments. These included sound detection measurement, otoacoustic emission and auditory brainstem response testing and speech perception evaluation. In addition, four infants (3–28 months) treated with disease-modifying therapies underwent auditory brainstem response assessment. Despite normal cochlear responses and normal sound detection, most child or young adult participants presented with clinically abnormal auditory neural function and significant speech perception deficits. Auditory brainstem response amplitudes were reduced and latencies increased relative to matched controls (*P* < 0.005) consistent with axonopathy and/or demyelination in the auditory brainstem. Furthermore, both monaural and binaural speech perception in noise were impaired (*P* < 0.01) suggesting the presence of significant neural distortion and spatial processing disruption. In contrast, evoked potential findings for our small group of pre-symptomatic infants were normal. The results of this study demonstrate that auditory neural and binaural processing deficits are common in children and young adults with SMA. As such, auditory assessment including auditory brainstem function and speech perception in background noise should be routinely carried out in this population. Interventions specifically designed to improve hearing in background noise (such as remote-microphone listening systems) should be considered to optimize speech/language and psychosocial development and academic outcomes in affected individuals.

## Teaser

Spinal muscular atrophy is a genetic disorder affecting 1 in 10 000 people. It mainly involves physical abnormalities (muscle weakness, etc.) but can also involve the sensory systems. This study identified hearing deficits severe enough to restrict communication and delay academic progress in most of the school-aged participants.

## Graphical Abstract

**Graphical Abstract:**
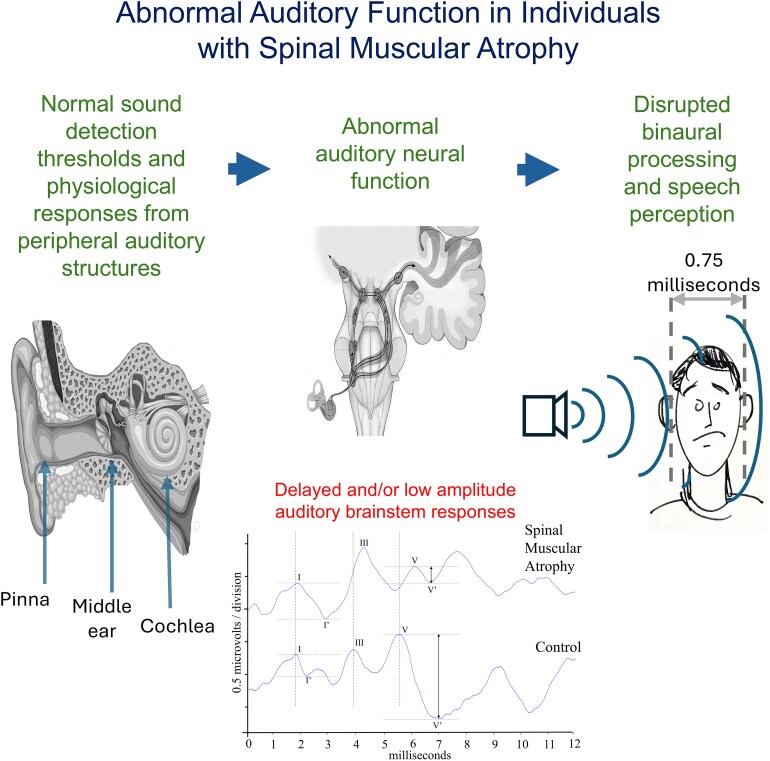

## Introduction

Spinal muscular atrophy (SMA) is an autosomal recessive disorder with an incidence of 1 in 10 000 that is characterized by hypotonia, progressive muscle weakness and atrophy.^1^ It is generally divided into three main subtypes (Types 1–3)^1^; however with the advent of newborn screening and implementation of pre-symptomatic disease-modifying therapies, the phenotypic classification of SMA is evolving.^2^ Approximately 95% of individuals with SMA have a homozygous deletion of the survival motor neuron protein 1 (*SMN1*) which encodes the SMN protein.^3^ Symptom severity is mediated by the number of copies of the paralogous *SMN2* gene which produces limited amounts of functional SMN protein and, as such, provides partial compensation of the lack of protein produced from *SMN1*.^4^

SMA primarily causes progressive loss of motor neurons in the spinal cord and lower cranial nerve nuclei but has also been associated with a sensory neuropathy and neuronopathy.^5-7^ Furthermore, evoked potential abnormalities (conduction delay and/or amplitude reduction) have been reported in multiple sensory systems including the somatosensory and visual pathways.^8^

Little is known about the hearing of individuals with SMA. No studies have reported auditory perceptual abilities, or even sound detection levels in affected patients. Abnormalities in auditory neural function have, however, been described in a limited number of cases. El Khateeb *et al*.^9^ presented a single paediatric subject with prolonged interpeak latencies on auditory brainstem response (ABR) assessment indicating delayed neural conduction between the vestibulocochlear nerve and lateral lemniscus.^10^ Similarly, Cheliout-Heraut and colleagues^8^ described ABR latency delays in 11 of 22 children with genetically confirmed SMA. In particular, these authors found that abnormalities were more frequent in children with SMA Type 1 than Type 2.

Evidence of auditory neural abnormality in SMA raises the possibility of functional hearing (speech perception) disruption. Perceptual deficits severe enough to impact speech/language acquisition, psychosocial development and academic progress have been described in infants with auditory neuropathies due to congenital/perinatal insult (including oxygen deprivation and hyperbilirubinaemia) as well as children and adults with progressive axonal and/or demyelinating conditions.^11^ Affected individuals show deficits in perceptual processes contingent on the precise neural representation of acoustic signals including discrimination of the brief timing cues that differentiate speech sounds, localization of sound sources and understanding of speech in background noise.^11-13^ The aims of this study were, therefore, to characterize the mechanisms underlying auditory dysfunction in SMA and to explore the concomitant functional hearing effects in a group of affected children and young adults.

## Materials and methods

Participants were recruited from the Royal Children's Hospital Melbourne Neuromuscular Clinic between 2022 and 2025. All children meeting the study selection criteria through this period were invited to take part. Inclusion criteria were (i) genetically confirmed SMA and (ii) ability to reliably complete auditory assessment.

Informed consent was obtained from all parents/guardians and age-appropriate assent was obtained prior to study participation. Project approval was obtained through the Ethics Committee of the Royal Victorian Eye and Ear and Hospital (# 07-747H-22).

### Auditory phenotyping

#### Sound detection thresholds

Behavioural hearing levels were established using a MAICO MA28 audiometer. Thresholds were obtained to pure-tone stimuli presented at octave frequencies between 250 and 8000 Hz. A four-frequency average level (4FA) based on hearing thresholds at the 500-, 1000-, 2000- and 4000-Hz test frequencies was calculated for each ear.

#### Otoacoustic emissions

Distortion product otoacoustic emission (DPOAE) assessment was performed bilaterally using the Intelligent Hearing Systems (IHS) Duet device. Stimulus tones ranged in frequency from 903 to 3623 Hz. Average response amplitude was calculated at 5 points across this range, and emissions were considered present if response amplitude was ≥6 dB above the noise floor for at least three consecutive test frequencies.^14^

#### Electrophysiology

ABRs were recorded to acoustic clicks presented at 90 dBnHL using the IHS Duet device. Electroencephalographic samples following 2000 stimuli were averaged to produce each test run, and two independent runs were compared to determine response repeatability. Waveform analysis was carried out by two experienced clinicians (blinded to group status) who determined response presence/absence, post-stimulus latency of Waves I, III and V and peak-to-peak amplitude of ABR Waves I and V. An amplitude ratio (Wave V/Wave I) was also calculated for each ear.

#### Monaural speech perception

Consonant-nucleus-consonant (CNC) word stimuli were presented via insert earphones using custom made (Australian native speaker) sound files saved to a portable laptop computer. The poorer hearing ear was assessed at 65 dBSPL (RMS)^15^ in two test conditions: quiet (target speech with no competing signal) and 0 dB signal-to-noise ratio (SNR) (both target speech and background noise at 65 dBSPL). The competing noise was continuous four-talker babble. A CNC list, consisting of 50 words, was presented in each condition and the participant was required to repeat each word. Responses were phonetically transcribed in real time, and the percentage of phonemes (speech sounds) correctly imitated was calculated.

#### Binaural speech perception

Assessment of binaural speech perception ability in background noise was carried out using the Listening in Spatialized Noise (LiSN-S) test run through a portable laptop computer.^16^ This assessment determines the subjects’ capacity to segregate target sentences from a competing speech noise. Participants were presented with 20–30 sentences and scored on the number of identified target words. Speech reception threshold (SRT) was established by varying the presentation level of the sentences relative to the background noise to determine the minimum SNR required to identify half of the target words. Reception threshold was established in four listening conditions which varied in terms of noise location (0^0^ versus 90^0^ azimuth) and vocal quality of speaker used to produce the target and background signals (i.e. same or different talker). The four conditions were DV90 (different voices spatially separated by 90^0^), SV90 (same voice separated by 90^0^), DV0^0^ (different voices from same direction) and SV0^0^ (same voice from same direction).

### Clinical data

Data on SMA type (Type 1, 2 or 3 or asymptomatic), *SMN2* copy number, age at symptom onset and treatment initiation were recorded. Motor function was assessed using validated motor outcome measures for SMA including the (i) Revised Hammersmith Scale which measures gross motor function in ambulant and non-ambulant children with SMA (scored between 0 and 69, with higher scores indicating better motor function),^17^ (ii) Children's Hospital of Philadelphia Infant Test of Neuromuscular Disorders (CHOP-INTEND) which measures gross motor function in non-ambulant infants and young children with SMA (scored between 0 and 64, with higher scores indicating better motor function)^18^ and (iii) the Egen Klassifikation 2 (EK2) which measures functional status in non-ambulant individuals with SMA (scored between 0 and 51 with lower score indicating better function).^19^ Motor measures performed within 6 months of auditory testing were included for school-aged children/young adults and within 3 months for infants.

### Statistical analysis

Auditory findings were analysed using the MINITAB-21 statistical program. All assumptions for parametric analyses were met. Group differences (SMA versus control data) were evaluated using paired *t*-tests. In each case, *t*- and *P*-values as well as 95% confidence ranges for the group difference were calculated. A significance level of *P* < 0.05 was used. Effect sizes (Cohen's *d*) were also generated. Outcomes for the SMA group were also subjected to χ² tests for association where normative clinical data was available. A linear mixed-model analysis was used to explore the relationship between key auditory measures and SMA demographics with participant as a random variable and age at assessment as a covariate. In this analysis, a Bonferroni correction was applied to control for Type 1 errors across multiple comparisons.

## Results

### Participant demographics

Twenty individuals (11 females) with genetically confirmed SMA were recruited to the study. Of these, 16 were school-aged children/young adults aged 6–20 years at assessment. Demographic details are shown in Table 1. A cohort of 16 age- and gender-matched individuals with no history of neurodegenerative disease served as controls for SMA participants aged 6 years and above. In each case, the age match was within 12 months. In addition, three pre-symptomatically treated infants diagnosed with SMA on newborn screening (aged 3–10 months at assessment) and one toddler with SMA Type 1 (aged 28 months at assessment) were evaluated (Table 1).

**Table 1:** Demographic data for study participants with SMA

Participant	Age (yrs)	SMA type	SMN2 copy number	Revised Hammersmith Scale	Ambulant	Age at treatment initiation (yrs)	Age at symptom onset (yrs)
SMA1	6.6	3	3	50	Yes	2.2	1.2
SMA2	8.5	2	3	19	No	4.7	0.6
SMA3	7.0	2	3	13	No	3.2	0.3
SMA4	13.1	3	3	50	Yes	8.9	1.4
SMA5	14.4	2	U	7	No	11.0	0.5
SMA6 ^a^	6.4	3	2	51	Yes	0.1	4.0
SMA7 ^a^	14.3	2	2	EK2 = 30	No	9.0	2.0
SMA8	14.2	3	4	69	Yes	14.3	14.0
SMA9	16.4	3	4	59	Yes	16.3	14.4
SMA10	13.7	ASx	4	69	Yes	Not on treatment ^b^	Not on treatment
SMA11	16.6	3	4	40	No	11.4	1.2
SMA12 ^c^	15.9	3	U	52	Yes	11.6	6.4
SMA13 ^c^	20.0	3	2	EK2 = 14	No	15.7	14.0
SMA14	12.0	2	U	EK2 = 17	No	6.6	1.5
SMA15	9.7	1	2	24	No	0.5	0.1
SMA16	5.1	1	2	51	Yes	0.3	0.2
SMA17	0.2	ASx	3	CHOP = 64	N/A	12 days	N/A
SMA18	0.3	ASx	3	CHOP = 61	N/A	11 days	N/A
SMA19	0.9	ASx	2	33	N/A	14 days	N/A
SMA20	2.3	1	2	6	No	0.2	0.1

Revised Hammersmith Scale is scored between 0 and 69, with higher score indicating better motor function.

ASx, asymptomatic; CHOP, Children's Hospital of Philadelphia Infant Test of Neuromuscular Disorders (CHOP-INTEND); EK2, Egen Klassification 2; N/A, not applicable; U, unknown.

^a^SMA6 and SMA7 are siblings who are compound heterozygous for a *SMN1* deletion and disease-associated sequence variant (c.683T>A, p.Leu288*). SMA6 was treated pre-symptomatically however developed symptoms and signs of SMA in early childhood.

^b^SMA10 was not eligible for treatment in Australia (pre-symptomatic with four copies of *SMN2*; is sibling of SMA11).

^c^SMA12 and SMA13 are siblings with homozygous disease-associated sequence variants (c.683T>A, p.Leu288*); previously reported by Li *et al*. (2024).^19^

### Clinical features

Three, five and eight children had SMA Types 1, 2 and 3, respectively. Four were asymptomatic; three of these were diagnosed on newborn screening (and treated with disease-modifying therapies) and one (SMA10) was tested during childhood due to an affected sibling. Nine children (excluding those under 24 months of age) were non-ambulant. Two siblings had homozygous disease-associated sequence variants in *SMN1*,^20^ and another sibling pair were compound heterozygous for a *SMN1* deletion and disease-associated sequence variant. Four children (SMA16, 18–20) had received gene therapy. Other treated participants were receiving nusinersen or risdiplam at the time of the study.

### Auditory findings in school-aged children and young adults

#### Sound detection thresholds

Behavioural hearing levels were normal bilaterally for each participant with SMA. Detection thresholds were within normal limits (≤20 dBHL) at each test frequency and four-frequency average levels were equivalent for SMA and control groups [SMA: 10.9 ± 5.8 dBHL; control: 9.0 ± 5.0 dBHL, *t*(31) = 1.37, *P* = 0.181, 95% CI: −0.9, 4.6 dB], and the effect size based upon Cohen's *d* classification was ‘small’ (*d* = 0.35).

#### Pre-neural cochlear responses

Each subject showed repeatable DPOAE responses indicating the presence of normal cochlear outer hair cell function in both ears.^21^ Mean response amplitude was equivalent for the SMA and control cohorts [SMA: 16.0 ± 5.8 dB; control: 14.9 ± 4.0 dB, *t*(31) = 0.80, *P* = 0.432, 95% CI: −1.7, 3.8 dB] and the effect size was ‘small’ (*d* = 0.22).

#### Auditory neural activity

**Figure 1:**
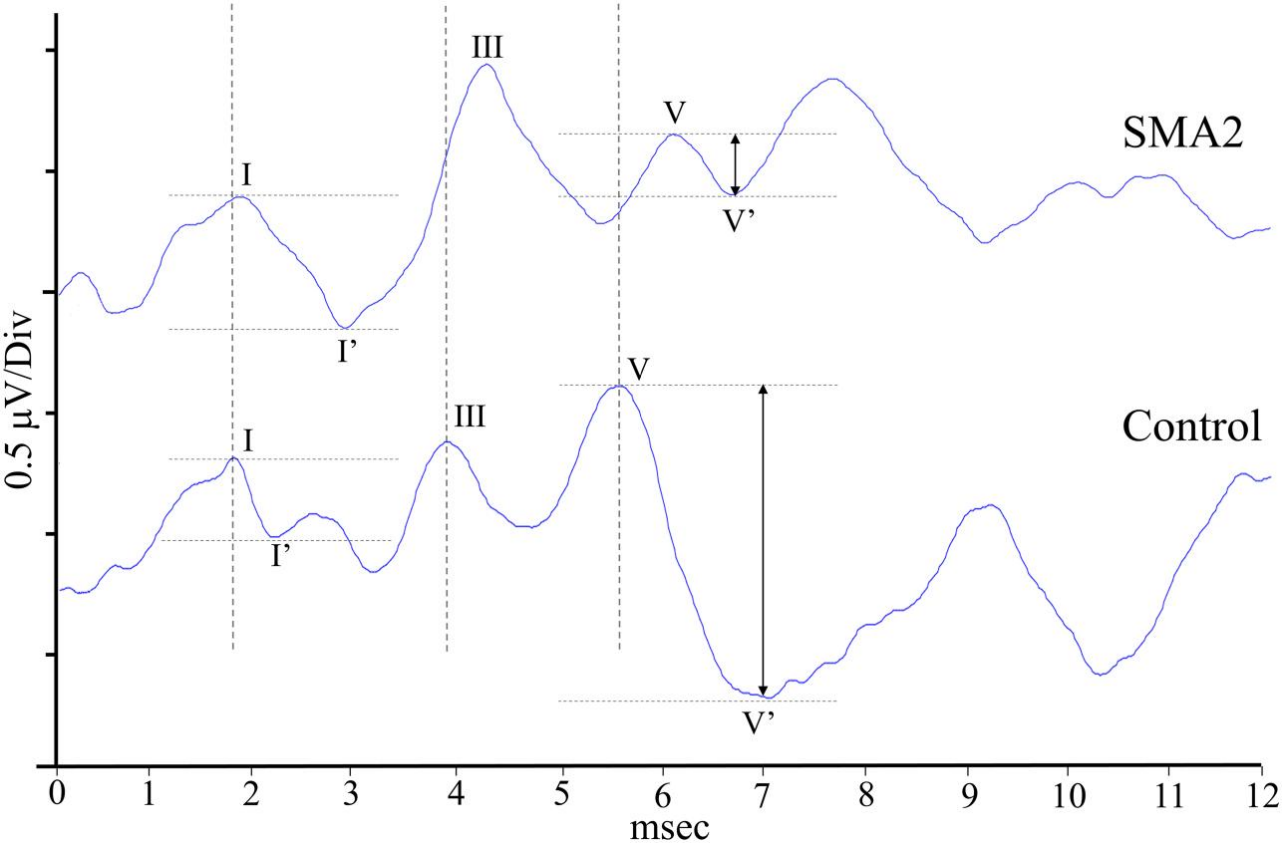
**ABRs for a child with SMA and his matched control**. ABR recordings for an 8-year-old child with SMA Type 2 (SMA2) (upper tracing) and his matched control participant (lower tracing) showing increased Wave III and Wave V latencies and reduced Wave V amplitude. The scale for the ordinate axis is 0.5 microvolts per division (µV/Div) and for the abscissa is milliseconds (ms). Wave I amplitude is defined as the difference (µV) between the positive peak in the 1.5–2.5 ms range (I) and the following negative peak (I′). Wave V amplitude is the difference between the positive peak in the 5–7 ms range (V) and the following negative peak (V′).

Repeatable ABRs were obtained bilaterally for each of the SMA participants. In most cases, however, neural activity in the VIIIth nerve and brainstem was abnormal. Seven of 16 individuals presented with response latencies outside published normative ranges^22^ representing a substantially higher rate of abnormality than expected for the general population (χ²: 50.6; *P* < 0.001). In addition, ABR amplitudes were significantly reduced in 13/16 participants (χ²: 195.8; *P* < 0.001). Figure 1 shows an example of the ABR waveforms for a typical SMA participant and his matched control.

Neural conduction velocity was lower in participants with SMA than controls. While there were no group differences for Wave I or Wave II post-stimulus latency, participants with SMA showed longer latencies for Wave III and Wave V (Table 2). Similarly, interpeak latencies (between Waves I–III, III–V and I–V) were all significantly longer in SMA participants (Table 2). Effect sizes were in the ‘medium’ to ‘large’ range based upon Cohen's *d* classification (Table 2).

**Table 2:** ABR findings for SMA and matched control participants

Participant group	Latency Wave I	Latency Wave II	Latency Wave III	Latency Wave V	Interpeak Latency I–III	Interpeak Latency III–V	Interpeak Latency I–V	Amplitude Wave I	Amplitude Wave V	Amplitude ratio V/I
SMA mean (SD)	1.66 (0.15)	2.65 (0.11)	4.00 (0.21)	5.89 (0.29)	2.34 (0.17)	1.90 (0.20)	4.23 (0.27)	0.40 (0.15)	0.47 (0.19)	1.22 (0.53)
Control mean (SD)	1.60 (0.16)	2.61 (0.12)	3.86 (0.20)	5.56 (0.18)	2.26 (0.13)	1.69 (0.12)	3.95 (0.16)	0.40 (0.18)	0.64 (0.23)	1.79 (0.83)
Difference mean (SD)	0.06 (0.27)	0.04 (0.21)	0.13 (0.30)	0.34 (0.40)	0.08 (0.15)	0.21 (0.22)	0.28 (0.27)	0.00 (0.23)	0.16 (0.29)	0.57 (0.83)
95% CI for paired difference	−0.04, 0.16	−0.04, 0.11	0.02, 0.24	0.19, 0.48	0.02, 0.13	0.12, 0.28	0.18, 0.38	−0.18, 0.08	−0.27, −0.06	−0.87, −0.27
*t*-value	1.22	0.86	2.53	4.77	2.79	5.25	5.90	0.02	−3.10	−3.89
*P*-value	0.233	0.398	0.017	<0.001	0.009	<0.001	<0.001	0.987	0.004	<0.001
Cohen's *d* (effect size)	0.39 (small)	0.26 (small)	0.68 (medium)	1.37 (large)	0.53 (medium)	1.27 (large)	1.26 (large)	0.0 (negligible)	0.81 (large)	0.82 (large)

Values for each parameter were based upon 32 data points from each group. Latency values (absolute and interpeak) are in milliseconds (ms). Amplitude values represent the peak-to-peak difference in microvolts (µV).

ABR response amplitude was also reduced in participants with SMA. Mean Wave I peak-to-peak amplitudes were equivalent in SMA and control groups, but Wave V peak-to-peak amplitude and Wave V/I ratios were significantly reduced for the SMA cohort. The effect size for Wave I amplitude was ‘negligible’, but was ‘large’ for Wave V and Wave V/I ratio (Table 2).

#### Speech perception in background noise

##### Monaural

Monaural speech perception in background noise was impaired in participants with SMA. While mean CNC phoneme scores for listening in quiet were equivalent for SMA and control groups, the SMA participants typically discriminated ∼10% fewer speech sounds than their matched counterparts in the presence of background noise. The effect size for the quiet condition was ‘small’, but it was ‘large’ for listening in background noise (Table 3).

**Table 3:** Speech perception findings for SMA and control participants

	Monaural		Binaural			
	CNC (quiet)	CNC (0 dBSNR)	DV90	SV90	DV0	SV0
SMA mean (SD)	94.6 (5.9)	34.4 (13.3)	−12.4 (3.5)	−10.8 (3.5)	−3.7 (2.6)	0.0 (1.8)
Control mean (SD)	96.6 (7.1)	44.0 (9.7)	−16.3 (2.5)	−13.4 (3.4)	−4.3 (1.7)	−0.6 (1.7)
Difference mean (SD)	2.0 (9.1)	9.6 (12.4)	3.9 (3.3)	2.6 (1.8)	0.6 (3.0)	0.6 (1.6)
95% CI for paired difference	−6.8, 2.9	−16.3, −3.0	2.1, 5.8	1.6, 3.6	−1.0, 2.3	−0.3, 1.5
*t*-value	−0.83	−3.01	4.59	5.43	0.84	1.41
*P*-value	0.396	0.007	<0.001	<0.001	0.416	0.179
Cohen's *d* (effect size)	0.3 (small)	0.83 (large)	1.3 (large)	0.76 (medium)	0.29 (small)	0.34 (small)

Monaural findings are % phonemes correctly imitated in monosyllabic words. Binaural results are mean SRTs (dB) for the Listening in Spatialized Noise (LiSN-S) test. Values for each parameter were based upon 16 data points from each group.

CNC, consonant-nucleus-consonant; DV90, different voices (target sentence and background) separated by 90°; DV0, different voices (target sentence and background), same direction; SV90, same voice (target sentence and background) separated by 90°; SV0, same voice (target sentence and background) same direction.

##### Binaural

Binaural speech perception was also affected. Eight of 15 participants with SMA presented with clinically abnormal perception (>2 SD lower than age-corrected means) [χ²: 73.8 (*P* < 0.001)]. Mean SRTs for conditions where target sentences and background noise emanated from different directions were 2.6–3.9 dB higher in the SMA group than for the matched control cohort. Effect sizes for these conditions were in the ‘large’ (DV90) and ‘medium’ (SV90) ranges (Table 3). This result indicates that the levels of noise needed to be significantly lower for listeners with SMA to correctly identify 50% of the target stimuli. In contrast, reception thresholds for conditions where target speech and noise emanated from the same direction showed no group differences. Effect sizes for these conditions were ‘small’. This result pattern, which is indicative of a spatial processing disorder, has been reported previously for patients with auditory neuropathies.^23^

#### Auditory measures and disease severity

There were no significant correlations between any of the key auditory findings [ABR amplitude ratio (Wave V/I), monaural speech perception in noise (CNC words 0 dBSNR) and binaural speech perception (DV90) condition] and SMN copy number, Revised Hammersmith Score, age of SMA symptom onset and disease duration [Bonferroni-corrected alpha level (*P* < 0.0167)]. SMA type, in contrast, correlated with ABR amplitude (*P* = 0.015) with those individuals presenting with the most severe SMA type (i.e. Type 1) showing the greatest degree of neural disruption. Correlations for SMA type and speech perception ability failed to reach significance (*P* > 0.0167) (Table 4).

**Table 4:** SMA demographics versus auditory measures (linear mixed-model analysis)

		SMA type		SMN2 copy number		Revised Hammersmith Score		Age at symptom onset		Disease duration	
		*N* = 16		*N* = 13		*N* = 16		*N* = 12		*N* = 12	
		Coefficient (SE)	*P*-value	Coefficient (SE)	*P*-value	Coefficient (SE)	*P*-value	Coefficient (SE)	*P*-value	Coefficient (SE)	*P*-value
Auditory brainstem response	Wave V/I amplitude	0.47 (0.17)	0.015	0.04 (0.21)	0.864	0.00 (0.01)	0.675	0.03 (0.03)	0.328	−0.03 (0.03)	0.331
Speech perception	CNC words (0 dBSNR)	10.2 (4.31)	0.034	−1.49 (4.47)	0.746	0.33 (0.18)	0.064	1.15 (0.73)	0.143	−1.20 (0.75)	0.134
	DV90	−1.83 (0.92)	0.072	−0.01 (0.45)	0.847	0.01 (0.02)	0.854	0.00 (0.00)	0.332	−0.16 (0.14)	0.269

Wave V/I amplitude: auditory brainstem response amplitude ratio (Wave V peak-to-peak/Wave I peak-to-peak). CNC words (0 dBSNR): monosyllabic words in background noise (0 dB SNR). DV90: SRT (LiSN-S test) different voices (target sentence and background) separated by 90°.

### Infants with spinal muscular atrophy

Each of the three pre-symptomatically treated infants and the toddler with SMA Type 1 (treated symptomatically) with SMA showed repeatable ABR waveforms to stimuli presented to the left and right ears. In each case, neural conduction velocity (interpeak latency) was within age-based normative ranges (Table 5).^22,24^ There are no age-based normative values for ABR amplitudes; however, the Wave V/I ratios all followed a similar pattern to that observed for the older controls, with Wave V peak-to-peak amplitudes greater than for Wave I (i.e. ratios > 1.0).

**Table 5:** ABR findings for infants with SMA

Participant	Age at assessment	Test ear	ABR latency (ms)						ABR amplitude
			Wave I	Wave III	Wave V	Wave I–III	Wave III–V	Wave I–V	Wave V/I ratio
SMA17	3 months	L	1.58	4.26	6.14	2.68	1.88	4.56	1.84
		R	1.58	4.27	6.07	2.69	1.80	4.49	1.25
	3 months average (95% range)		*	*	*	2.67 (2.37–2.97)	2.24 (1.75–2.74)	4.76 (4.34–5.18)	*
SMA18	4 months	L	1.51	4.15	6.20	2.64	2.05	4.69	1.15
		R	1.64	4.17	6.32	2.53	2.06	4.59	1.64
	4 months average (95% range)		*	*	*	2.63 (2.33–2.93)	2.21 (1.69–2.73)	4.69 (4.27–5.11)	*
SMA19	11 months	L	1.64	4.03	6.32	2.39	2.29	4.68	1.48
		R	1.64	4.09	6.26	2.45	2.17	4.62	1.64
	11 months average (95% range)		*	*	*	2.48 (2.18–2.78)	2.12 (1.60–2.64)	4.46 (4.04–4.88)	*
SMA20	28 months	L	1.89	4.01	6.07	2.12	2.06	4.18	1.50
		R	1.90	4.20	6.14	2.30	1.94	4.24	1.62
	28 months average (95% range)		*	*	*	2.40 (2.10–2.70)	1.99 (1.49–2.49)	4.34 (3.92–4.76)	*

Latency values (absolute and interpeak) are in milliseconds (ms). Amplitude values represent the peak-to-peak difference in microvolts (µV). Normative data adapted from Chiappa *et al*. (1979) and Issa and Ross (1995). *No normative data available.

## Discussion

This study revealed auditory neural abnormalities and significant functional hearing deficits in our population of school-aged children/young adults with SMA. Despite presenting with normal cochlear physiologic responses (OAEs) and normal sound detection, most participants had clinically abnormal ABRs and more than half suffered speech perception deficits in background noise severe enough to restrict communication in everyday listening conditions and impede psychosocial and academic progress. In contrast, auditory neural responses in a small group of pre-symptomatic infants treated with disease-modifying therapies were normal.

Abnormal auditory neural activity was identified in most participants with SMA. In particular, 13/16 individuals from our child/young adult cohort showed reduced ABR amplitudes (Wave V and Wave V/I ratio) compared to published norms^22^ and matched controls. That is, synchronized activity in the central brainstem was reduced relative to that of the distal portion of the vestibulocochlear nerve. This result has not been reported previously in people with SMA but is common in patients with other neurodegenerative diseases affecting the auditory neural pathways including axonal forms of Charcot-Marie-Tooth disease (CMT Type 2), Friedreich ataxia and neurofibromatosis Type 1.^11^ The magnitude of amplitude loss seen in in this study is comparable to that seen in CMT Type 2.^25^

There are two pathophysiological mechanisms that may underpin ABR amplitude reduction. The first is axonopathy. Loss of auditory axons and ganglion cells results in de-afferentiation or a reduction in the amount of neural activity available to contribute to the evoked potential. Although SMA is primarily a motor neuronopathy, reports of dorsal root ganglia degeneration^7^ and sensory axonal neuropathy^6^ in infants with SMA Type 1 raise the possibility of a primary auditory axonopathy in SMA. Neuropathologic descriptions of auditory pathway involvement in SMA are limited. While early post-mortem studies reported abnormalities of the cranial nerve VIII nucleus in infants with SMA prior to the discovery of the *SMN1* gene,^26^ subsequent studies have identified abnormalities of the medial geniculate nucleus in severe prenatal/neonatal forms of the disease.^27^

A second possible mechanism for ABR amplitude reduction is dys-synchrony. Scalp-recorded evoked potentials are extracted from the background EEG via an averaging technique. This algebraic summation process requires that the timing of the neural response to each individual stimulus be almost identical. Disruption of the synchrony of neural activity may occur as a secondary consequence of axonopathy due to irregular conduction speeds in damaged axons or secondary demyelination due to axonal damage.^28,29^

Additionally, primary demyelination, distinct from demyelination secondary to axonal loss, was suggested in participants with SMA who showed evidence of severely delayed auditory neural conduction. Seven of 16 individuals presented with clinically abnormal interpeak latencies and, on average, ABR conduction times were 0.3 ms longer than those of matched controls. Post-stimulus latencies for Wave I and Wave II were unaffected indicating normal activity in the auditory nerve. Wave I–III interpeak intervals were, however, prolonged indicating delayed activation at the level of the cochlear nuclei. Furthermore, delays were evident between Waves III–V consistent with conduction abnormality between the cochlear nucleus and lateral lemniscus.^10^ Similar degrees of Wave I–III latency prolongation have been reported for patients with demyelinating forms of Charcot-Marie-Tooth Type 1 disease (CMT Type 1).^25,30^ However, Wave III–V interpeak latencies are relatively preserved in CMT Type 1 compared to our cohort, suggesting the presence of both central (brainstem) and peripheral auditory pathway demyelination in SMA. Disorders with central nervous system demyelination such as multiple sclerosis^31^ show delayed central conduction in the auditory brainstem, with prolonged Wave III–V interpeak latencies. Although it is difficult to prove a primary demyelinating process in SMA, histological studies have demonstrated demyelination in sensory tracts and peripheral nerves^5,7^ and *in vitro* and animal models indicate that SMN is required for peripheral and possibly central nervous system myelination.^32-34^

The primary functional effect of disrupted auditory neural activity is impaired perception of complex acoustic signals—such as speech. Disruption of the neural code distorts the representation of subtle timing cues in the speech signal and the degree of this distortion is correlated with perceptual ability.^12,13^ This affects speech discrimination in quiet listening conditions and particularly in background noise where listeners are less able to use brief quiet periods in the noise to access the speech information.^35^ In the current study, monaural speech perception ability for school-aged and adult participants was unaffected in quiet, but in noise was severely impaired relative to matched controls. For speech presented at a 0 dB SNR (simulating conditions in a typical school classroom,^36^ participants with SMA could only identify ∼approximately one of the three phonemes in each stimulus word—a perceptual level insufficient for speech understanding).

Disruption of auditory neural firing patterns also affects binaural processing as temporal distortion in each auditory nerve means that signals cannot be effectively combined in the brainstem and central pathways.^23,37^ As a consequence, affected listeners are less able to judge the location of sound sources and differentiate between target speech and background noise when they arise from different directions.^37^ This process, known as ‘spatial streaming’, requires the perception of subtle inter-aural cues such as the brief (≈50 µs) timing differences occurring as the signal reaches one ear before the other. The phenomenon affords listeners with normal binaural processing a 10–15 dB release from the masking effect of background noise and is consistently diminished in individuals with auditory neuropathies.^23^ In the current study, 8 of the 15 participants with SMA showed evidence of clinically abnormal binaural processing and mean SRTs were 2.5–4.0 dB poorer than for matched controls when target speech and background noise were spatially separated (Table 3). As an increase in classroom noise of only 0.5 dB is sufficient to affect academic outcomes, this degree of impairment represents a significant threat to developmental progress in school-aged children.^38^

The degree of auditory neural disruption in our SMA cohort was considerably less than reported previously. Where our group showed mean ABR I–V latency delays of ≈0.3 ms, Cheliout-Heraut *et al*.^8^ found conduction delays of 0.7–0.9 ms, albeit in a group of children with much greater disease severity—the 11 children in this study with SMA Type 1 were all ventilator dependent and tetraplegic, and of the 11 with SMA Type 2, the best motor function was sitting. In comparison, we present individuals with a range of SMA types and, in some cases, mild to no symptoms (Table 1). Notably, all four asymptomatic children (three treated infants and one untreated child) with SMA had normal auditory function. This raises the possibility that measures of auditory function may reflect overall disease severity/progression and may have a role as biomarker.

Correlations between audiology findings and SMA characteristics only found relationships between SMA type and ABR amplitude and SMA type and monaural speech perception in noise (Table 4). No correlations were found between auditory function and *SMN2* copy number, disease duration or motor outcome scores. These inconsistent correlations are likely attributable to the small sample size and incomplete data for some variables, which represent important study limitations. Another possible limitation is that we did not consider the participants’ language or cognitive abilities as potential confounds. These factors are unlikely to have affected the sound detection or evoked potential findings but could have adversely affected the speech perception results.^39^ The fact that the SMA participants scored as well as matched controls on sentence testing for conditions where there were no spatial cues (DV0 and SV0) does, however, indicate that they both understood the activity and had sufficient language skills to complete the task.^16^

A further study limitation is the lack of participant self-assessment data. While it is well established that impaired speech perception-in-noise affects communication-, psychosocial- and academic development in children,^40^ further work might investigate the real-world hearing/communication experiences of individuals with SMA.

The strength of this study is the detailed hearing protocol capable of detecting subtle monaural and binaural processing deficits—at least in children able to complete the behavioural speech testing (i.e. 6 years and above).^16^

## Summary and recommendations

In conclusion, this study demonstrates auditory neural abnormalities and clinically relevant hearing deficits in most of the school-aged children and young adults with SMA in this study. Audiometric thresholds were normal in all cases despite the presence of significant ABR and speech perception deficits, indicating that standard audiologic testing (which is typically restricted to sound detection assessment) would fail to detect these abnormalities. Larger studies are required to confirm the findings from this study, which has important management implications, particularly in light of the increasing awareness of broader language and cognitive delays in children with SMA.^41-43^

The focus of this project was children and young adults with SMA, but future work should look at older individuals with the condition. This is particularly important as the addition of peripheral hearing loss (which is common in older adults) to the auditory processing deficits we have identified would likely exacerbate functional hearing and communication difficulties in this population.^44^

Management of patients with auditory neural deficit is challenging. Amplification (via conventional hearing aids) is typically not appropriate as perception is limited by the degree of neural distortion rather than audibility so the listener is presented with a louder, but equally unclear signal.^11^ Interventions specifically designed to improve hearing in background noise such as personal remote-microphone systems^45^ may have a positive impact. These devices prioritize the signal from the primary speaker by recording the voice via a microphone near the mouth (usually at the lapel) and transmitting it directly to an earpiece worn by the listener.

In the era of newborn screening and pre-symptomatic/early treatment with disease-modifying therapies, it will be important to monitor audiology findings to determine if auditory neural abnormalities and hearing deficits are also abrogated by early treatment.

## Data Availability

The data will be shared with suitably qualified researchers upon request.
